# The impact of right ventricular lead position on outcomes in cardiac resynchronization therapy patients

**DOI:** 10.3389/fcvm.2026.1719908

**Published:** 2026-02-17

**Authors:** Yanfei Wang, Yan Xiong, Rana Abdul Qadir, Chunchang Qin, Enrun Wang, Guodong Chen, Zhaoyan Li, Lingyu Zhang, Fengpeng Jia, Yijia Tang

**Affiliations:** 1 Department of Cardiovascular Medicine, The First Affiliated Hospital of Chongqing Medical University, Chongqing, China; 2 Department of Cardiology, Sichuan Provincial People’s Hospital, School of Medicine, University of Electronic Science and Technology of China, Chengdu, China

**Keywords:** cardiac resynchronization therapy, clinical outcome, efficacy, heart failure, right ventricular electrode location

## Abstract

**Background:**

The effect of right ventricular (RV) lead position on the response to cardiac resynchronization therapy (CRT) remains unclear. We evaluated the effects of different RV lead positions on electrophysiology, echocardiography, and clinical outcomes.

**Methods:**

This was a retrospective cohort study. A total of 253 patients received CRT with left ventricular (LV) leads implanted in the LV posterolateral coronary vein were included in this study. According to the position of RV lead, the patients were divided into low septal (LSP) group (141 cases), medium septal (MSP) group (36 cases), high septal (HSP) group (32 cases) and left bundle branch area pacing (LBBAP) group (44 cases). The primary endpoint included a composite of rehospitalization for heart failure (HF) and all-cause mortality, assessed using Kaplan–Meier and Cox proportional hazards analyses. Secondary endpoints included changes in CRT response, arrhythmic events, device-related complications, pacing parameters, QRS duration, and echocardiographic parameters at 12-month follow-up.

**Results:**

There were no statistically significant differences in baseline characteristics among the four groups. The non-response rate of CRT (defined as failure to achieve an increase in LVEF >10% and an improvement in NYHA class by at least 1 grade) in the LSP group (48.2%) was higher than that in the HSP group (34.4%), MSP group (16.7%) and LBBAP group (18.2%) (*P* < 0.008), and the risk of ventricular arrhythmia was the highest (*P* = 0.003). QRS wave shortening and LV reverse remodeling were significantly greater in MSP and LBBAP groups than in LSP and HSP groups (*P* < 0.05). During a mean follow-up of (22.7 ± 4.4) months, the composite endpoint of heart failure rehospitalization and all-cause death did not differ significantly among the four groups (*P* > 0.05).

**Conclusion:**

RV middle septum or left bundle branch area pacing may improve electrical synchronization, reverse ventricular remodeling, and reduce the incidence of non-response to CRT and arrhythmia in patients with heart failure receiving CRT.

## Introduction

Cardiac Resynchronization Therapy (CRT) has proven to be an effective treatment for patients with chronic heart failure (HF) and prolonged QRS duration who have not responded adequately to pharmacological therapy (1). CRT achieves mechanical synchronization by aligning the electrical activity of the left and right ventricles, thereby improving heart function and reducing hospitalization rates and mortality (2). Despite its success, approximately one-third of patients fail to benefit from CRT (3). Traditionally, the response to CRT was evaluated by changes in left ventricular ejection fraction (LVEF) or left ventricular end-diastolic volume (LVEDV). However, using left ventricular remodeling alone to assess the clinical benefit of CRT has limitations. As HF is a progressive condition, stabilization of LV function and improvement in clinical status should also be considered as therapeutic success. Thus, the failure to respond to CRT may be overestimated based solely on these metrics. Nevertheless, non-response to CRT remains a significant challenge in clinical practice (4).

Several factors influence the efficacy of CRT, including the etiology of HF, improper pacing parameters, and suboptimal lead placement (5–8). Previous studies have underscored the importance of LV lead positioning on CRT outcomes, demonstrating its significant impact on both efficacy and occurrence of adverse events. However, there is limited research on the effects of right ventricular (RV) lead placement, and the findings are inconsistent (9, 10). Historically, RV lead placement has been at the apex. Recent evidence suggests that this non-physiological implantation may lead to long-term adverse cardiac effects, such as valve regurgitation and ventricular remodeling (11). Studies have shown that RV septal positioning may offer better CRT efficacy and superior cardiac ultrasound parameters compared to the RV apex (10, 12). Despite growing evidence of the benefits of non-apical RV lead placement, the optimal septal position remains unclear. Research on how different septal positions influence the efficacy and adverse reactions of CRT is still lacking.

In addition, recent developments in left bundle branch area pacing (LBBAP) have highlighted its potential in improving the symptoms of HF. LBBAP has been shown to provide superior cardiac resynchronization compared to traditional biventricular pacing (13). The implantation of an RV lead within the left bundle branch area (LOT-CRT) to improve the outcomes of conventional CRT has not been well studied.

The aim of this study was to evaluate the impacts of various RV lead positions on lead parameters, QRS duration, echocardiographic measures, and clinical outcomes in patients receiving CRT.

## Methods

### Study population

Patients who underwent CRT implantation in the Department of Cardiology at Sichuan Provincial People's Hospital and the First Affiliated Hospital of Chongqing Medical University from May 2021 to January 2024 were included. Inclusion criteria for patients were as follows: age ≥18 years, ineffective optimal drug therapy for 3 months before implantation, QRS duration ≥130 ms or proportion of predicted ventricular pacing >40%, New York Heart Association (NYHA) functional class II-IV, LVEF ≤35%, and at least 1 year of follow-up with echocardiography and device follow-up. The exclusion criteria for patients were as follows: patients lost to follow-up, patients with CRT upgrade, patients with implantable cardioverter defibrillators, patients with persistent atrial fibrillation (AF), patients without LV lead implantation in the posterolateral or lateral cardiac coronary veins. The enrolled patients were divided into the RV low-septal pacing group (LSP), mid-septal pacing (MSP) group, high-septal pacing (HSP) group, and left bundle branch area pacing (LBBAP) group.

This was a retrospective cohort study. It is in accordance with the Helsinki Declaration and has been approved by the Medical Ethics Committee of Sichuan Provincial People's Hospital [batch number: Lunyan (Research) 2022 No. 448] and the First Affiliated Hospital of Chongqing Medical University (batch number: K2023-539).

### Baseline characteristics and echocardiography

Clinical data include gender, age, medical history, NYHA classification, laboratory data, electrocardiogram, echocardiography, electrode parameters and positions. Patients underwent transthoracic echocardiography 1 year before and after CRT implantation. Simpson disk method was used to measure LVEF, LVEDD and LAD in apical four-chamber and two-chamber views using the Vivid E95 transthoracic echocardiography device manufactured by General Electric Medical Systems (USA) Co, LTD with M5Sc probe. CRT response was evaluated according to the change of LVEF during follow-up. CRT response was defined as an increase in LVEF >10% and a decrease in NYHA class of at least 1 grade (14).

### CRT procedural, localization of RV lead positions and pacing parameters

In this study, we chose the transvenous approach and used commercially available leads and devices. The most suitable coronary sinus branch was selected, and the LV electrode (2-pole or 4-pole) was sent into the appropriate site through the sheath tube. The electrode parameters and diaphragm stimulation were tested, and finally the LV electrode was fixed. Subsequently, the spiral active electrodes were implanted into different parts of the right atrium and right ventricle. LBBAP was implanted and parameters were tested according to the method and relevant criteria reported by Huang Weijian et al. (15), using C315 sheath and 3,830 solid cavity electrode from Medtronic company or Selectra 3D sheath and Solia hollow steel wire supported spiral electrode from Biotronik Company. Finally, the images were stored under the right anterior oblique, left anterior oblique and anteroposterior x-ray projection positions. The implanting position was decided by pacing doctors.

**Figure 1:**
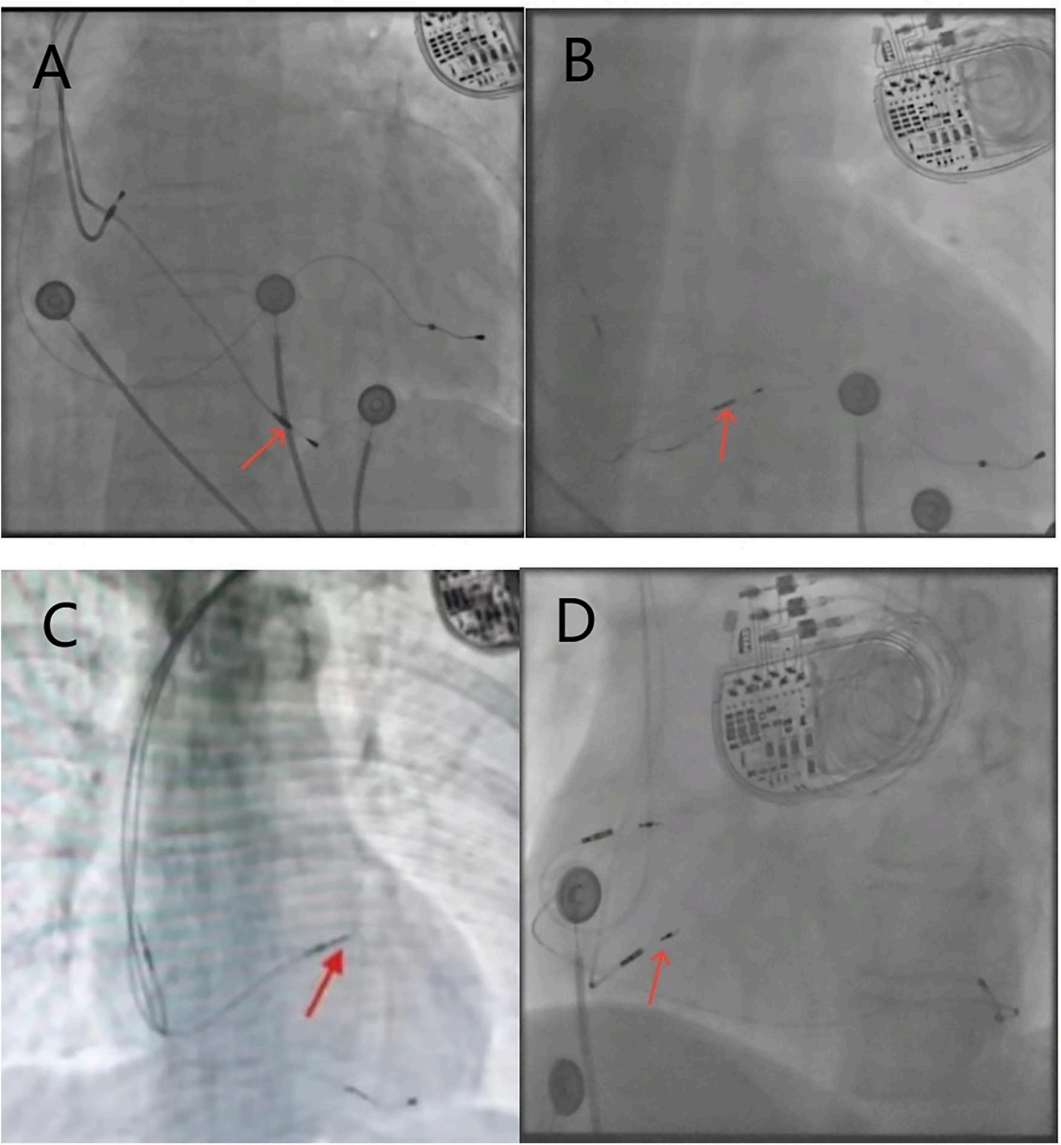
X-ray fluoroscopy images of the right ventricular electrode position, with red arrows indicating the implantation site of the RV electrode. **(A)** Posteroanterior view of the low-septum; **(B)** posteroanterior view of the mid-septum; **(C)** posteroanterior view of the high-septum; **(D)** left anterior oblique view of the left bundle branch area.

We used objective measurement criteria based on x-ray images rather than subjective anatomical description during operation to classify RV lead positions, aiming to establish a unified and repeatable classification system to minimize grouping bias. The positions of the lead in the RV septum were confirmed by the relative position of the x-ray anterior-posterior view of the heart silhouette and vertebral body shadow. The heart silhouette was divided into three regions: high, middle and low, to determine the relative height of the RV electrode tip within the heart silhouette. High-septum, the distance from the bottom of the heart silhouette is more than 2 vertebral bodies. Mid-septum, the distance from the bottom of the heart silhouette is 1.5–2 vertebral bodies. Low-septum, the distance from the bottom of the heart silhouette is less than 1.5 vertebral bodies (Figure 1).

Based on the left anterior oblique (LAO) view, the RV electrode was implanted into the LBBA, and the sheath tube was adjusted so that the electrode was oriented perpendicular to the ventricular septum ((Figure 1D). Right bundle branch block (RBBB) was observed during pacing, and bundle branch potential was locally recorded. The interval between pacing signal and R-wave peak in V5 or V6 remained constant and <75 ms. Local angiography showed that the tip of the electrode reached the left ventricular septal surface.

**Figure 2:**
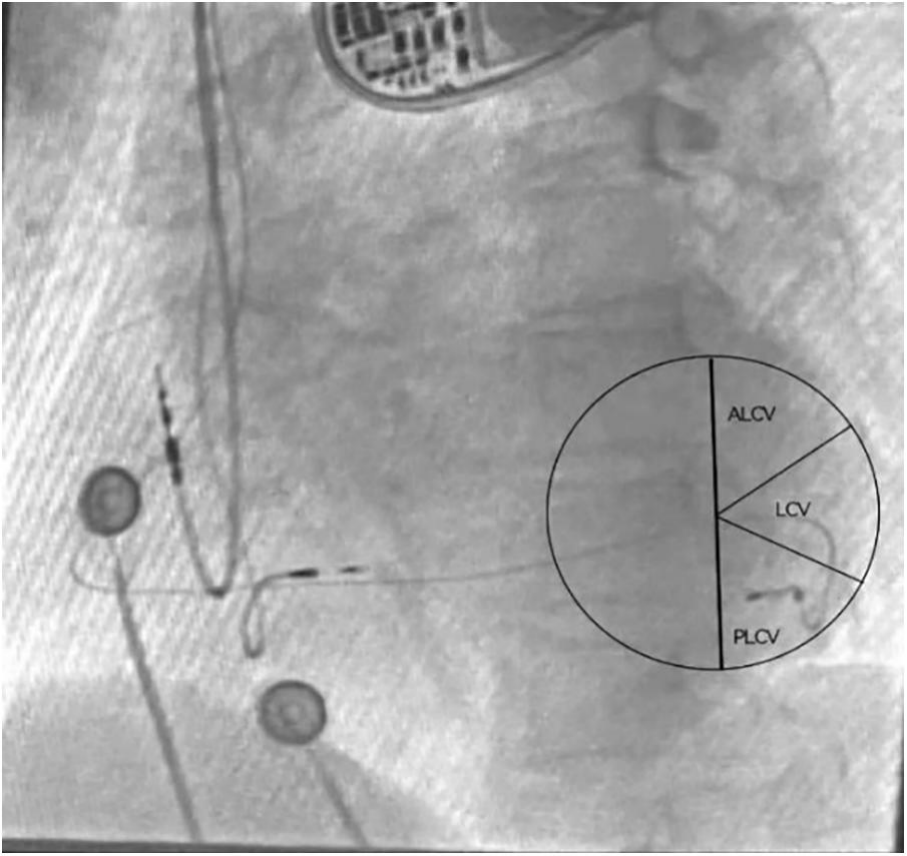
Locating the left ventricular lead position through perspective imaging. The left ventricular electrode was implanted in the PLCV under the LAO 30°view. ALCV, Anterolateral cardiac vein; LCV, Lateral cardiac vein; PLCV, Posterolateral cardiac vein.

To control for the confounding effect of left ventricular (LV) lead position, only patients with the LV lead positioned in the posterolateral or lateral cardiac coronary vein, as confirmed by the left anterior oblique (LAO) view (Figure 2), were included in the study (14).

All imaging data of left and right ventricular lead positions were interpreted by two cardiologists who were unaware of the group assignments. If the results were controversial, the results were interpreted by a third cardiologist. If the results were still controversial, the case was excluded from the study.

### Follow-up and definitions of endpoints

Routine clinical follow-up was performed within 1 year after CRT implantation, including clinical improvement, electrocardiogram, echocardiography and RV lead pacing parameters changes. Adverse events such as lead dislocation, infection of the pacing system, abnormal increase of threshold and abnormal sensing were recorded during the follow-up period. The primary endpoint was a composite of HF rehospitalization and all-cause death during 2 years of follow-up. The secondary endpoints were CRT response, ventricular tachyarrhythmia (including ventricular tachycardia and ventricular fibrillation), atrial fibrillation, pacemaker-related complications, QRS duration changes and echocardiographic parameters changes. HF rehospitalization was defined as worsening, hospitalization time > 24 h, and the need for intravenous diuretics. All-cause death was defined as death from any causes that occurred during the follow-up period. CRT response is considered as LVEF improvement > 10% and NYHA classification reduction of at least 1 grade (14). Abnormal changes of RV pacing lead parameters were defined as pacing threshold > 3.0 V, sensing ≤5.0 mV, impedance > 2,000 Ω or < 300 Ω. Changed echocardiographic parameters including LVEDD, LAD and LVEF defined as changes before and after CRT implantation. All arrhythmic events and pacemaker parameters were determined by two electrophysiologists.

### Statistical analysis

The statistical software used was IBM SPSS Statistics 26.0. Measurement data with normal distribution were expressed as mean ± standard deviation (x ± s), and analysis of variance was used for comparison between groups. Nonparametric test was used for non-normal distribution data. Count data were expressed as frequency (percentage), and comparison between groups was analyzed using the chi-square test or Fisher exact probability. Wilcoxon rank sum test was used to compare the ranked data. Time to the primary composite end point was described with the use of the Kaplan–Meier method and compared with the use of the log-rank test. Cox regression analysis was used to calculate the Hazard ratio (HR) and 95% Confidence interval (CI) to evaluate the relationship between RV lead location and the primary endpoints. For the comparison of count data between groups, a Bonferroni-corrected significance level of *P* < 0.008 (0.05/6 pairwise comparisons) was applied to control for the family-wise error rate. For all other analyses, *P* < 0.05 was considered statistically significant.

## Results

A total of 321 patients were enrolled in this study, RV lead position data of 253 patients were available for analysis, of which 141 (55.7%) RV leads were located in low septum, 36 (14.2%) in middle septum, 32 (12.6%) in high septum, and 44 (17.4%) in LBBA. 68 patients were excluded because of left ventricular lead placement in an anterior or who were lost to follow-up. The mean age was 66.2 ± 10.3 years, 103 (40.7%) were female, the mean LVEF was 27.5 ± 5.3%, and the mean QRS width was 157.7 ± 15.7 ms. All patients received optimal medical therapy for HF.

### The baseline clinical characteristics

There were no significant differences in gender, age, basic medical history, cardiac function classification, laboratory indexes, electrocardiogram, RV lead parameters, procedure-related complications, and postoperative medication among the four groups. In the LBBA group, operation and x-ray exposure time were slightly longer than the other three groups, and the difference were statistically significant (Tables 1, 2).

**Table 1:** The clinical baseline data were compared among the four groups.

Baseline data	LSP (*n* = 141)	MSP (*n* = 36)	HSP (*n* = 32)	LBBAP (*n* = 44)	*P*
Age (years)	65.4 ± 10.2	69.3 ± 10.6	64.7 ± 8.8	67.2 ± 11.0	0.163
Gender (male, %)	93 (66.0)	17 (47.2)	17 (53.1)	23 (52.3)	0.104
Diabetes (%)	33 (23.4)	12 (34.3)	10 (31.3)	11 (25.0)	0.530
Hypertension (%)	34 (24.1)	16 (44.4)	11 (34.4)	12 (27.3)	0.096
Kidney disease (%)	25 (17.7)	7 (19.4)	6 (18.8)	8 (18.2)	0.996
COPD (%)	11 (7.8)	4 (11.1)	4 (12.5)	2 (4.5)	0.572
PAH (%)	10 (7.1)	6 (16.7)	2 (6.3)	2 (4.5)	0.260
AST (U/L)	49.2 ± 77.1	46.1 ± 49.9	35.3 ± 22.3	41.0 ± 21.5	0.685
ALT (U/L)	56.1 ± 141.9	34.5 ± 35.8	30.3 ± 21.7	32.3 ± 29.0	0.408
Cr (umol/L)	101.0 ± 85.1	95.2 ± 38.9	115.8 ± 166.6	96.7 ± 41.5	0.775
eGFR [mL/(min × 1.73 m^2^)]	73.8 ± 26.0	65.9 ± 21.8	70.5 ± 23.6	71.6 ± 22.9	0.402
BNP (pg/mL)	1,788.2 ± 3,490.6	1,638.1 ± 1,684.4	1,230.8 ± 1,057.5	791.8 ± 893.6	0.191
NYHA (%)					0.377
II	47 (33.3)	15 (41.7)	14 (43.8)	12 (27.3)	
III	94 (66.7)	21 (58.3)	18 (56.3)	32 (72.7)	
Preoperative rhythm					
AF (%)	23 (16.3)	7 (19.4)	7 (21.9)	3 (6.8)	0.268
VAs (%)	27 (19.1)	4 (11.1)	6 (18.8)	7 (15.9)	0.706
LBBB (%)	96 (68.6)	27 (77.1)	22 (68.8)	35 (79.5)	0.447
Postoperative medications					
Beta-blockers	120 (85.1)	31 (86.1)	30 (93.8)	34 (77.3)	0.236
ACEI/ARB	41 (29.1)	7 (19.4)	9 (28.1)	6 (13.6)	0.168
Spironolactone	110 (78.0)	31 (86.1)	23 (71.9)	38 (86.4)	0.308
Furosemide	122 (86.5)	33 (91.7)	29 (90.6)	38 (86.4)	0.776
Nocinol	88 (62.4)	21 (58.3)	17 (53.1)	34 (77.3)	0.136
SGLT-2	31 (22.0)	8 (22.2)	4 (12.5)	8 (18.2)	0.646

Values are given as percent of patients or mean + SD. COPD, chronic obstructive pulmonary disease; PAH, pulmonary arterial hypertension; AST, aspartate aminotransferase; ALT, alanine aminotransferase; Cr, creatinine; eGFR, estimated glomerular filtration rate; BNP, B-type natriuretic peptide; AF, atrial fibrillation; VAs, ventricular arrhythmias; NYHA, New York Heart Association; LBBB, left bundle branch block; ACEI, angiotensin-converting enzyme inhibitors; ARB, angiotensin II receptor blockers.

**Table 2:** CRT operative data were compared among the four groups.

Surgical-related data	LSP (*n* = 141)	MSP (*n* = 36)	HSP (*n* = 32)	LBBAP (*n* = 44)	*P*
Duration of operation (min)	130.0^△^ (127.0,133.0)	130.0^△^ (126.3,134.0)	130.5^△^ (127.3,135.8)	140.5 (138.3,142.8)	<0.001
X-ray exposure time (min)	25.0^△^ (22.0,28.0)	25.0^△^ (22.0,28.0)	25.0^△^ (22.3,27.8)	29.0 (27.3,31.0)	<0.001
Surgical complications					
Pneumothorax (%)	1 (0.7)	0	0	0	0.429
Pocket hematoma (%)	0	1 (3.1)	1 (2.8)	0	0.496
Electrode dislocation (%)	0	0	0	1 (2.3)	0.080
Intraoperative right ventricular electrode parameters					
Threshold (V)	0.68 ± 0.25	0.76 ± 0.26	0.75 ± 0.17	0.71 ± 0.30	0.266
Impedance (*Ω*)	630.3 ± 137.5	640.3 ± 123.9	656.3 ± 122.5	670.5 ± 113.3	0.303
Perception (mV)	14.0 ± 4.7	12.6 ± 5.7	14.1 ± 5.6	12.6 ± 4.6	0.205

Compared with the left bundle branch area group.

△ *P* < 0.05.

### The changes of electrocardiogram and echocardiography

**Figure 3:**
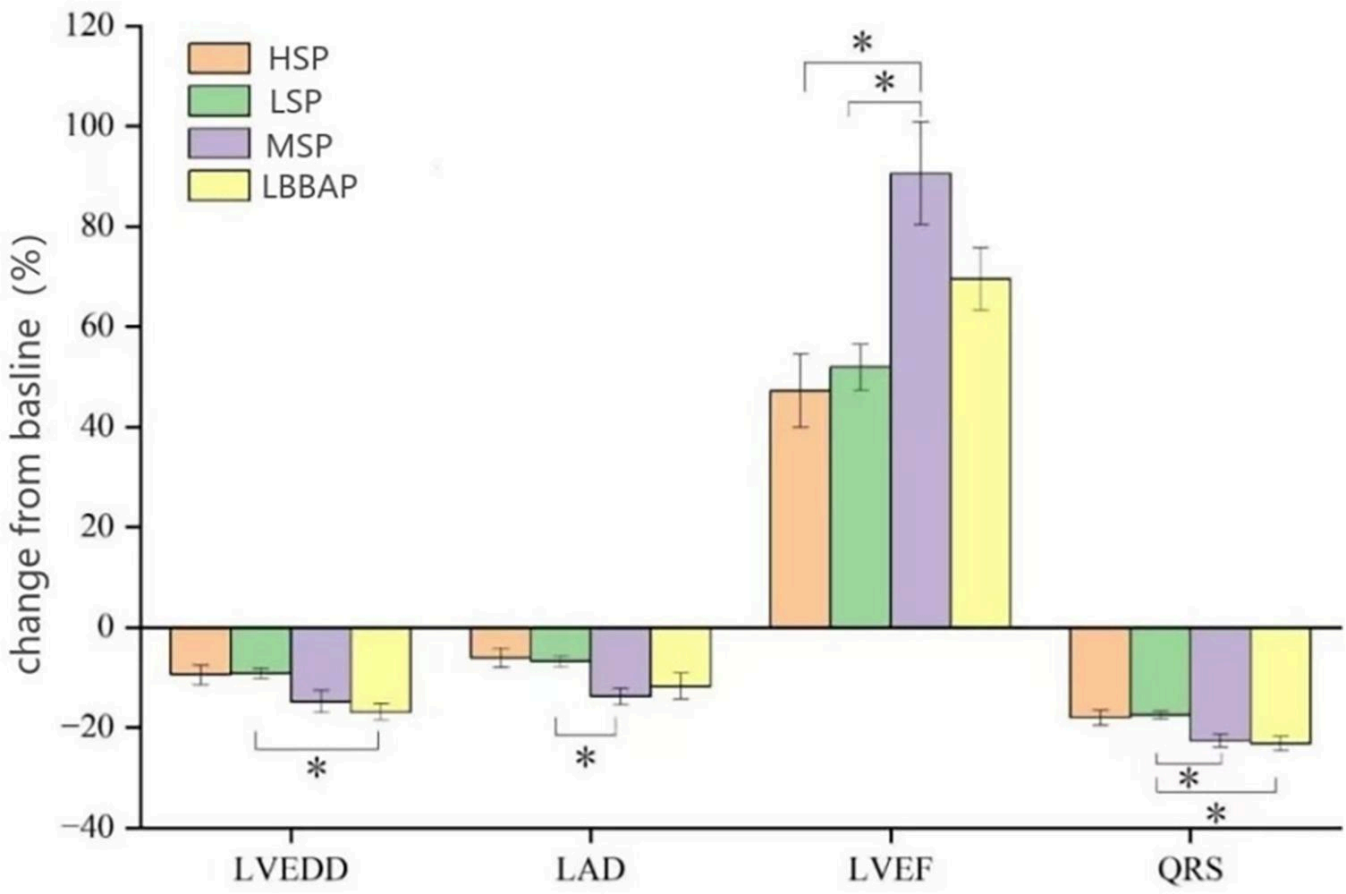
LV reverse remodeling and changes in QRS duration from baseline [absolute changes (%) in LVEDD, LVD, LVEF and QRSd] were compared among the four groups. LVEDD, left ventricular end diastolic diameter; LAD, Left atrial diameter; LVEF, left ventricular ejection fraction; QRSd, QRS duration. *P < 0.05.

The QRS duration in each group after operation was significantly shorter than that before operation (*P* < 0.05). Compared with the degree of QRS wave shortening in the LSP and HSP (−17.5 ± 9.1%; −18.0 ± 8.8%), MSP and LBBAP had a more obvious changes (−22.7 ± 7.8%; −23.2 ± 9.1%, *P* < 0.05). Patients in MSP and LBBAP had a significant decreased LVEDD and LAD after procedure compared with LSP and HSP groups (*P* < 0.05). LVEF increased after operation in all four groups (*P* < 0.05), and the patients with leads in MSP and LBBAP had a more changes than patients with LSP and HSP (*P* < 0.05) (Table 3 and Figure 3).

**Table 3:** Echocardiographic parameters and QRS duration were compared among the four groups.

Variables	LSP (*n* = 141)	MSP (*n* = 36)	HSP (*n* = 32)	LBBAP (*n* = 44)	*P*
LVEDD (mm)					
Before implantation	67.6 ± 8.4	64.8 ± 7.1	66.1 ± 8.4	66.3 ± 7.1	0.241
1 year after implantation	61.3 ± 11.1^*△#^	55.0 ± 9.2*	59.6 ± 8.8*	54.5 ± 7.2*	<0.001
Change from baseline (%)	−9.2 ± 12.4^△^	−14.8 ± 13.2	−9.5 ± 11.1	−16.9 ± 11.3	0.001
LAD (mm)					
Before surgery	45.7 ± 7.3	45.4 ± 6.3	47.9 ± 7.5	43.8 ± 6.6	0.112
1 year after implantation	42.4 ± 8.2*	39.1 ± 6.7^*&^	44.7 ± 6.7^*△#^	39.4 ± 5.9^*&^	0.002
Change from basline (%)	−6.8 ± 12.7#	−13.8 ± 10.1	−6.1 ± 10.8	−11.8 ± 17.2	0.008
LVEF (%)					
Before surgery	27.2 ± 5.2	26.6 ± 5.7	28.6 ± 5.6	28.6 ± 4.8	0.180
1 year after implantation	40.1 ± 12.5^*△#^	49.3 ± 14.2*	41.8 ± 12.6*	47.7 ± 10.6*	<0.001
Change from baseline (%)	51.9 ± 55.6#	90.6 ± 61.7^&^	47.2 ± 41.1#	69.5 ± 41.1	<0.001
QRSd (ms)					
Before surgery	157.8 ± 17.1	160.7 ± 13.6	157.8 ± 15.1	155.0 ± 12.7	0.456
One year after surgery	129.4 ± 13.1^*△^	123.6 ± 9.2*	128.7 ± 13.9^*△^	118.6 ± 13.5^*&^	<0.001
Change from basline (%)	−17.5 ± 9.1^△#^	−22.7 ± 7.8	−18.0 ± 8.8	−23.2 ± 9.1	<0.001

LVEDD, left ventricular end diastolic diameter; LAD, left atrial diameter; LVEF, left ventricular ejection fraction; QRSd, QRS duration. Compared with before operation in the same group. %change from basline=(postoperative indicator–preoperative indicator)/preoperative indicator.

* *P* < 0.05; compared with left bundle branch area.

△ *P* < 0.05; compared with mid septum group.

# *P* < 0. 05; compared with high septum group.

& *P* < 0. 05.

### Associations of RV lead location with clinical outcomes

**Figure 4:**
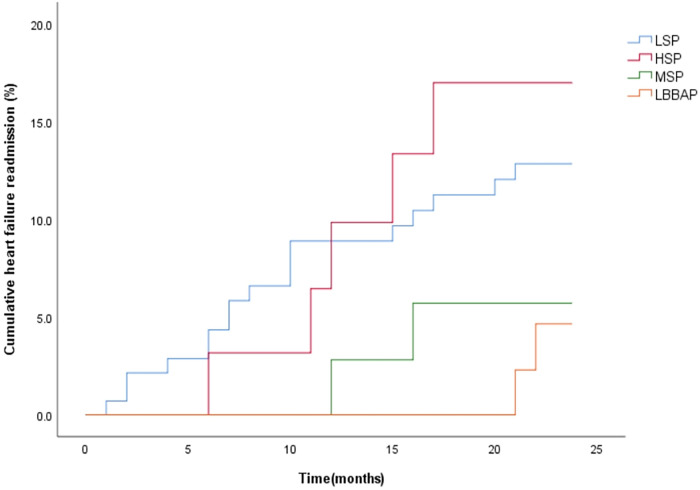
Kaplan–Meier curves were used to compare the time estimates of HF rehospitalization/all-cause death among the four groups with different RV lead positions. (logrank *p* = 0.233).

**Figure 5:**
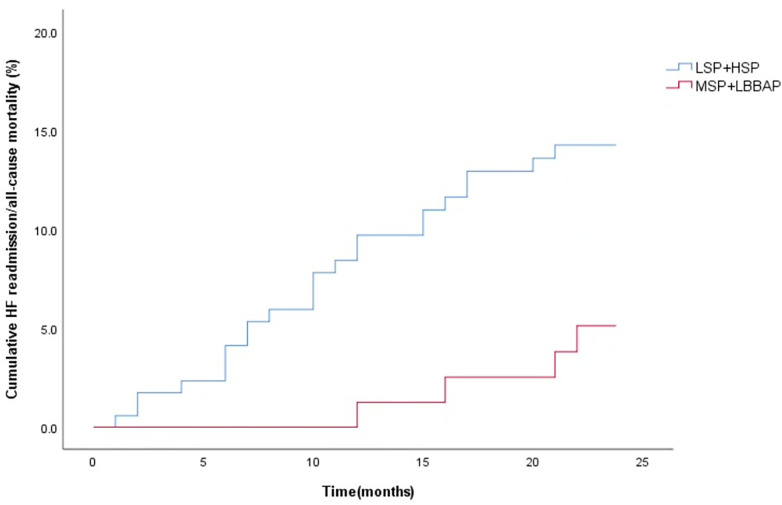
Kaplan–Meier curves were used to compare the time estimates of HF rehospitalization/all-cause death among the LSP + HSP group and MSP + LBBAP group. (logrank *p* = 0.044).

During the 12-month follow-up, there were no significant differences in RV lead parameters, device-related complications and incidence of AF among the four groups (*P* > 0.008). However, LSP had a relatively higher risk of ventricular arrhythmia (35.5%) and a higher CRT non-response rate (48.2%, *P* < 0.008) (Table 4). During a mean follow-up of 22.7 ± 4.4 months, the cumulative incidence of HF rehospitalization/all-cause death (*P* = 0.233) was similar among the four groups (Figure 4). However, the cumulative incidence of HF rehospitalization/all-cause death was found to be statistically different between the LSP + HSP group and the MSP + LBBAP group (*P* = 0.044, Figure 5). The LSP and HSP groups were associated with earlier HF rehospitalization and all-cause death. However, cox multivariate analysis showed that there were no association between the RV lead position and the risk of HF rehospitalization/all-cause death (*P* > 0.05) (Tables 5, 6).

**Table 4:** Comparison of the incidence of adverse events among the four groups.

Variables	LSP (*n* = 141)	MSP (*n* = 36)	HSP (*n* = 32)	LBBAP (*n* = 44)	*P*
Electrode dislocation (%)	0	0	0	1 (2.3)	0.080
Pacing system infection (%)	1 (0.7)	0	0	0	0.429
Right ventricular electrode parameters one year after surgery					
Threshold (V)	0.76 ± 0.18	0.81 ± 0.18	0.78 ± 0.16	0.82 ± 0.45	0.403
Impedance (*Ω*)	514.9 ± 91.4	505.6 ± 81.8	542.9 ± 80.1	538.3 ± 100.4	0.164
Perception (mV)	12.9 ± 5.7	11.4 ± 5.9	12.4 ± 5.4	11.4 ± 4.3	0.277
Abnormally elevated threshold (%)	0	0	0	1 (2.3)	0.080
Abnormal electrode impedance (%)	1 (0.7)	0	0	0	0.429
Abnormal electrode sensing (%)	8 (5.7)	3 (9.4)	3 (8.3)	2 (4.5)	0.794
Ventricular arrhythmias (%)	50 (35.5)*	5 (13.9)	9 (28.1)	5 (11.4)	0.003
Atrial fibrillation (%)	30 (21.3)	7 (19.4)	7 (21.9)	9 (20.5)	0.994
CRT non-response rate (%)	68 (48.2)^*△^	6 (16.7)	11 (34.4)	8 (18.2)	<0.001
Heart failure readmission (%)	17 (12.1)	2 (5.6)	5 (15.6)	2 (4.5)	0.231
All-cause mortality (%)	6 (4.3)	0	3 (9.4)	0	0.042
Heart failure readmission and all-cause mortality (%)	18 (12.8)	2 (5.6)	5 (15.6)	2 (4.5)	0.197

Chi-square tests for pairwise comparisons within four groups, *P* values have been Bonferroni adjusted. compared with left bundle branch area.

* *P* < 0.008; compared with mid septum group.

△ *P* < 0. 008.

**Table 5:** Risk of all-cause death and HF readmission in the 4 groups during 2 years of follow-up.

Grouping	HR^a^	95%CI	*P*
LSP			
MSP	0.578	0.101–3.308	0.538
HSP	1.259	0.364–3.352	0.716
LBBAP	0.544	0.120–2.477	0.431

a Models were adjusted for age, sex, creatinine, eGFR, QRS width and morphology, LV, LVEF, hypertension, diabetes, history of renal disease, and COPD.

**Table 6:** Risk of all-cause death and HF readmission in the LSP + HSP group and MSP + LBBAP group during 2 years.

Variables	LSP + HSP	MSP + LBBAP	HR^a^	95%CI	*P*
Heart failure readmission (%)	22 (12.7)	4 (5.0)	0.560	0.172–1.826	0.336
Heart failure readmission and all-cause mortality (%)	23 (13.3)	4 (5.0)	0.535	0.168–1.705	0.290

a Models were adjusted for age, sex, creatinine, eGFR, QRS width and morphology, LV, LVEF, hypertension, diabetes, history of renal disease, and COPD.

## Discussion

We report 3 major findings in this study. First, LBBAP were associated with slightly longer procedure and fluoroscopy time than other three RV septal pacing positions, but the four groups had similar very lower complications and long-tern stable pacing parameters. Second, compared to LSP and HSP, MSP and LBBAP were associated with significantly greater reductions in QRS duration, more pronounced left ventricular reverse remodeling (reflected by improvements in LVEF and reductions in LVEDD, and a lower incidence of ventricular arrhythmias. Third, although no statistically significant differences were observed in all-cause mortality and HF rehospitalization among the groups, further grouping of RV lead positions suggested that LSP plus HSP were associated with earlier occurrence of adverse events, indicating a potentially worse prognosis.

### Procedural complications and pacing parameters

Compared with routine RV septal pacing positions such as LSP, MSP and LSP, LBBAP required more complex manipulations and precise measurements. Additionally, patients receiving CRT always had enlarged right atrium or right ventricle, which increased the difficulties of approaching target and acquiring the ideal parameters. Meanwhile, operators need a longer time of learning curve to be familiar with LBBAP. All of the above brought with slightly longer procedure and fluoroscopy times. However, there were no differences at procedural complications and device-related adverse events. During the 2 year follow-up, the four pacing positions kept excellent stable pacing parameters and impedence.

### Impact on QRS duration, arrhythmias and ventricular remodeling

Our results showed that LBBAP and MSP significantly shortened QRS duration compared with LSP and HSP. These findings partially align with prior studies, such as an Indian cohort which reported that both MSP and HSP significantly narrowed QRS duration compared to apical pacing (16). However, our study showed there was a heterogeneity in the HSP group patients. In some cases, the RV lead traversed the supraventricular crest and entered the right ventricular outflow tract (RVOT), creating a non-physiological transmural excitation pattern, which caused the wide QRS duration. This may explain the suboptimal QRS wave narrowing observed in our HSP group. We found that LSP was associated with a higher risk of postoperative ventricular arrhythmias, but the incidences of AF were similar among the four groups.This harmful ventricular events may be due to the unfavorable shortening QRS wave duration.

The superior electrical and mechanical performance of LBBAP may be attributed to its ability to recruit the native conduction system, thereby achieving more physiological ventricular activation and reducing electrical dyssynchrony (17). Furthermore, by narrowing the QRS complex and optimizing electromechanical synchrony, LBBAP effectively minimizes the risk of adverse remodeling and arrhythmogenesis (18, 19). Our results showed that LBBAP and MSP significantly shortened QRS duration and improved indices of reverse remodeling, including increased LVEF and reduced LVEDD, compared with LSP and HSP.

LBBAP demonstrating robust electrical synchronization and excellent echoing findings, which supports the emerging utility of LOT-CRT **(**20–22). However Parale et al. reported limited benefit of LOT-CRT on QRS narrowing (23), this may reflect underlying conduction heterogeneity or myocardial fibrosis, particularly in patients with lateral wall conduction delay,extensive scar burden and diffused ventricular conduction block (24). These findings highlight the importance of individualized anatomical and electrophysiological assessment when considering conduction system pacing.

Moreover, the link between QRS duration, mechanical synchrony, and CRT response further supports the concept of electromechanical coupling. This dyssynchrony of low and high RV septal pacing exacerbate adverse remodeling, leading to delayed lateral wall contraction and paradoxical septal motion, perpetuating an “electromechanical vicious cycle.” Our findings reinforce prior literature (16, 25, 26) and suggest that both QRS duration and echocardiographic parameters should be used as complementary markers in evaluating CRT efficacy.

### The impact on HF rehospitalization and all-cause mortality

Although MSP and LBBAP achieved better performances on the changes of electrophysiology and cardiac remodelling, we did not observe the significant differences among the four groups in all-cause mortality/HF re-hospitalization during the 24 month-follow up after procedure. Further analysis suggests that the cumulative incidence of HF re-hospitalization/ all-cause mortality in the LSP + HSP was higher than that in the MSP + LBBAP.This difference may arise from the following three aspects. First, after optimizing the groups, the baseline characteristics were more balanced, enhancing the comparability between groups and reducing the interference of confounding factors on the results, thereby reducing the probability of type II errors caused by sample heterogeneity.Second, the two year-follow up is relatively short time so that there was not an obvious difference of the primary endpoint.Third, we observed that some patients receiving CRT without pronounced left ventricular reverse remodeling or shortening QRS duration showed significant improvement of HF symptoms or stable blood pressure, which provided the opportunity to optimize the medicine therapy. Those patients also had a lower rate of re-hospitalization and all-cause mortality, in spite of non-response on CRT by traditional definition.

Therefore, although the overall clinical endpoint differences did not reach a significant level, optimizing the right ventricular lead position still suggested that choosing a better pacing site may improve electrical synchrony and ventricular remodeling, ultimately translating into clinical benefits.

### Study limitations

This study is a retrospective study. To our knowledge, there have been relatively few studies on the impact of different RV lead positions on the therapeutic effect of CRT patients. It is worth noting that we only observed the impact of RV lead positions on the electrical remodeling and cardiac structure changes in CRT patients, but the evidence of the main endpoints events was insufficient. Several factors may account for this. First, the limited number of patients and relatively short follow-up period may have underpowered the study for detecting differences in long-term outcomes. Second, a disproportionate number of patients were assigned to the LSP group, largely due to operator preference and simple skills. Third, the position of right ventricular lead was confirmed by x-ray fluoroscopy.Chest CT scan may be more accurate than our present method. Future research should aim to combine anatomical imaging with real-time electrical delay measurements to form a multiple navigational strategy, which is expected to further standardize the procedure and improve the overall response rate of CRT.

## Conclusion

This study highlights the role of RV lead positioning in determining the clinical efficacy of CRT. Both MSP and LBBAP may be associated with superior outcomes on QRS during narrowing, echocardiographic improvement, and arrhythmia reduction. Future research should focus on integrating advanced imaging and electrophysiological mapping to guide individualized lead positioning. Larger, multicenter trials with extended follow-up are essential to validate the long-term prognostic outcomes of different RV lead locations.

## Data Availability

The original contributions presented in the study are included in the article/Supplementary Material, further inquiries can be directed to the corresponding authors.
